# Practical Notes on Diagnosis and Treatment

**Published:** 1907-02-09

**Authors:** 


### Practical Notes on Diagnosis and Treatment.

### General Tuberculosis.

General tuberculosis in childhood is a very rapid disease, being usually of a month or six weeks' duration.

### Anæmia and Phthisis Pulmonalis.

The early detection of tubercular mischief in anæmic subjects is of vital importance, for in my experience the disease in such persons tends to advance rapidly.—*Dr. J. Edward Squire.*

### Chancre of the Tonsil.

The diagnosis of chancre of the tonsil is usually attended by some elements of difficulty since the sore may be a secondary one. If it be a chancre it ought to be on one side only, with a glandular bubo on one side only of the neck, and there ought to be a clear history of its having preceded by some weeks the eruption on the skin.—*Mr. Jonathan Hutchinson.*

### Ergot in Migraine.

Dr. W. H. Thomson advises ergot in migraine and also in neuralgias which recur periodically. He orders 1 drachm of the liquid extract to be taken in water as soon as the pain is noticed, and the patient is then advised to remain in the recumbent posture. In migraine three such doses are taken at intervals of an hour; in neuralgia the drug is given thrice daily.

### Toothache.

The following is recommended to relieve the pain of a carious tooth:—Extract of opium, camphor, of each 5 parts; balsam of Peru and mastiche, of each 1 part; chloroform, 10 parts. To be used to plug the cavity.

### Eclampsia in Pregnancy.

As to how to *treat the pregnancy* in a bad eclampsia, opinions differ. I strongly advise you to induce labour, and if labour is in progress to accelerate it judiciously. The use of full doses of morphine ($\frac{1}{3}$ to $\frac{1}{2}$ grain) hypodermically we owe to Professor Veit. Should the fits persist after the first dose, another should be given in a few hours' time. It should not be given when there is coma.—*Dr. Berry Hart.*

### Thyroid Gland in Myxœdema.

The dose for a patient with myxœdema or cretinism must be very much smaller than when the drug is given for other diseases. A dose which would be absolutely without effect on a healthy man may in these patients produce profound constitutional disturbance. When once a cure has been obtained, a bi-weekly, weekly, or even fortnightly dose is sufficient to maintain a proper condition of health.—*Dr. Hector Mackenzie.*

